# Gene expression of psychiatric disorder-related kinesin superfamily proteins (*Kifs*) is potentiated in alternatively activated primary cultured microglia

**DOI:** 10.1186/s13104-024-07078-y

**Published:** 2025-01-30

**Authors:** Suguru Iwata, Mitsuhiro Hyugaji, Yohei Soga, Momo Morikawa, Tetsuya Sasaki, Yosuke Takei

**Affiliations:** 1 Department of Anatomy and Neuroscience, Institute of Medicine, University of Tsukuba, 1-1- 1, Tennodai, Tsukuba, Ibaraki 305-8577 Japan; 2 College of Biological Sciences, University of Tsukuba, 1-1-1, Tennodai, Tsukuba, Ibaraki 305- 8572 Japan; 3 College of Medicine, School of Medicine and Health Sciences, University of Tsukuba, 1-1-1 Tennodai, Tsukuba, Ibaraki 305-8577 Japan

**Keywords:** Microglia, Kinesin superfamily proteins (KIFs), Alternatively activated microglia, Primary culture, Interleukin-4, Real-time qPCR, Psychiatric disorder

## Abstract

**Objective:**

Reactivity of microglia, the resident cells of the brain, underlies innate immune mechanisms (e.g., injury repair), and disruption of microglial reactivity has been shown to facilitate psychiatric disorder dysfunctions. Although cellular analyses based on cultured microglia have been conducted, the molecular mechanism regulating microglial polarization remains elusive. We established a primary microglia culture that enabled manipulation of the substate of cells. This allowed us to investigate the expression levels of psychiatric disorder-related *Kifs* messenger RNA (mRNA) in each condition. *Kifs* encode molecular motor proteins that transport cargo along microtubules, which are thought to dynamically reorganize during a substate change.

**Results:**

As a candidate for a crucial *Kifs* gene that is associated with microglia polarization, we selected psychiatric disorder-related *Kifs* including *Kif17*. We found that the relative amounts of *Kif3a*, *Kif17*, and *Kif13a* mRNA were potentiated in alternatively activated microglia, whereas there were no significant changes in activated microglia. Furthermore, the microglia derived from a mouse line which possesses a mutation inducing truncated KIF17 indicated disrupted morphological phenotype of alternatively activated microglia. These results suggest that the potentiation of specific molecular motor expression is required to maintain the function of alternatively activated microglia.

**Supplementary Information:**

The online version contains supplementary material available at 10.1186/s13104-024-07078-y.

## Introduction

Microglia are resident immune cells in the brain that originate from a yolk sac and are crucial for brain development, neuronal function, and brain injury repair [1–4]. In response to extrinsic cues derived from the induction of inflammation or injury, microglia in a steady state activate and extensively alter gene expression and morphology [5, 6]. Reactive microglia are often categorized as either activated microglia or alternatively activated microglia. Activated microglia are associated with pro-inflammatory mediators and induce inflammation and neurotoxicity, whereas alternatively activated microglia are associated with anti-inflammatory and repairing activity and secrete neurotrophic factors. It has been also reported that overactivated microglia contribute to brain damage and neurodegeneration [1, 7]. Recently, primary microglia cultures, which maintain ramified cells, have been established [8], revealing that the transition between homeostatic and reactive states induces drastic reorganization of the microtubule cytoskeleton [9]. However, the molecular mechanism underlying the functionality of transformed microglia following cytoskeleton remodeling remains elusive.

Kinesin superfamily proteins (KIFs) are microtubule-based motor proteins that transport various types of cargo to specific functional areas. This intracellular transport system is essential for cellular morphogenesis, function, and survival [10, 11], and each KIF has specific cargos and transport mechanisms. Notably, KIFs that transport receptors related to synaptic plasticity, such as the *N*-methyl-d-aspartate receptor (NMDAR), have been reported to be involved in not only the delivery of essential supplies, but also the regulation of the local neuronal physiological state [12–14]. Additionally, these KIFs are associated with the pathogenesis of various psychiatric disorders, such as schizophrenia (SCZ) and autism [15–21]. Therefore, we examined the messenger RNA (mRNA) expression levels of psychiatric disorder-related *Kifs* in each microglial substate and the morphology of microglia stimulated by interleukin (IL)-4 which possess a mutation inducing truncated KIF17.

## Materials and methods

### Mice

Male and pregnant female C57BL/6J mice were purchased from Japan CLEA (Tokyo, Japan). Mice were housed in a 12-h light/dark cycle environment with free access to food and water. Newborn mice for cultures were decapitated, immediately after inducing hypothermic anesthesia by putting them on an ice bath. This method was used for minimization of animal suffering.

A *Kif17* mutation mouse line was established using the clustered regularly interspaced short palindromic repeat (CRISPR)–CRISPR-associated protein 9 system [22, 23] by the Takahashi laboratory (University of Tsukuba) as previously reported [14]. Our plan was to introduce the Y576X mutation into *Kif17* gene (Additional file 1: Fig. S1a). To facilitate the detection of the genotypes, we engineered the donor vector to contain XhoI restriction site downstream of the mutation (Additional file 1: Fig. S1b). The px330 vector containing guide sequence and the donor vector containing the Y576X mutation were injected into mouse zygotes, and then forty-three pups were generated. Genomic DNA from the pups were confirmed not to integrate the vectors, and then directly sequenced. As a result, we identified two mice that carried the mutation and an intact *Kif17* gene upstream of the mutation. *Kif17* mutation mice were genotyped by PCR and subsequent digestion by KpnI and XhoI. The genotyping primers are the following: 5′-ATTCTCAAGGCGGAAGTCCT-3′ and 5′-GTCTGGGATGAGTCCGACAT-3′. The genotypes were identified as shown in Additional file 1: Fig. S1c.

### Primary microglia culture

Primary microglia cultures were prepared as described previously [8, 9] with minor modifications. Briefly, cerebral cortices were removed from pups (postnatal day 1–3) and subsequently chopped and dissociated with 0.25% trypsin (#15090-046, Gibco) for 40 min at 37 °C, followed by gentle trituration. The cells were suspended in Dulbecco’s Modified Eagle Medium (DMEM, #11965-092, Gibco), supplemented with 10% fetal bovine serum (#S1400, Biowest) and GlutaMAX I (#35050, Gibco), plated in 80 cm^2^ cell culture flasks (#153732, Thermo Fisher Scientific), and cultured for 13–15 days. At confluence, culture flasks were tapped several times to detach and collect microglial cells and plated on cover glasses with sterile-filtered homeostatic microglial culture medium, which comprised DMEM/F12 (#21041-025, Gibco), containing 100 units/mL penicillin-streptomycin (#15140122, Gibco), 5 µg/mL N-acetyl cysteine (#A9165, Sigma-Aldrich), 5 µg/mL insulin (#I6634, Sigma-Aldrich), 100 µg/mL apo-transferrin (#T1147, Sigma-Aldrich), 100 ng/mL sodium selenite (#S5261, Sigma-Aldrich), 2 ng/mL human transforming growth factor-β2 (TGF-β2, #100-35B, Peprotech), 100 ng/mL murine IL-34 (#577602, BioLegend), and 1.5 µg/mL ovine wool cholesterol (#700000P, Avanti Polar Lipids). Twenty-four hours after plating, the cells were treated with 100 ng/mL lipopolysaccharide (LPS, #L4391, Sigma-Aldrich) and 20 ng/mL interferon-γ (IFNγ, #315-05, Peprotech) or with 20 ng/mL IL-4 (#214-14, Peprotech) to induce the activated or alternatively activated phenotype, respectively. Subsequently, the cells were fixed or eluted 48 h after stimulation.

### Immunocytochemistry

Primary cultured microglia were fixed with 4% paraformaldehyde for 10 min. Fixed microglia were permeabilized with 0.1% Triton X-100, blocked with 5% bovine serum albumin, and incubated with a rabbit anti-ionized calcium-binding adapter molecule 1 (Iba-1) polyclonal antibody (#019-19741, Fujifilm) at 4°C overnight. The samples were then incubated with goat anti-rabbit Alexa Fluor^®^ 488 (#A11034, Invitrogen) at room temperature for 1 h while 4’,6-diamidino-2-phenylindole (DAPI, #340–07971, Dojindo Laboratories) staining was performed simultaneously. All images were acquired using a TCS SP8 confocal laser scanning microscope (Leica, Hesse, Germany). Morphology analyses were performed by quantitatively measuring the cell area, solidity (expressed as the ratio of the convex area to the cell area), and maximum span across the convex hull (MSACH; maximum distance between two points across the convex hull) using the ImageJ software (National Institutes of Health, MD, USA).

### Reverse-transcriptase-quantitative polymerase chain reaction (RT-qPCR)

Total RNA recovery and RT-qPCR were conducted as described previously [14]. The complementary DNA samples were subjected to amplification with GeneAce SYBR™ qPCR Mix II (#313–09423, NIPPON GENE) using a QuantStudio 5 real-time PCR system (Applied Biosystems, MA, USA), with pre-incubation at 95 °C for 10 min, denaturation at 96 °C for 10 s, annealing at 54–58 °C for 15 s, and an extension step at 60 °C for 1 min for a maximum of 100 cycles. The PCR primers are listed in Table 1. The results from *Gapdh* were treated as internal control.

**Table 1:** PCR primers used in the study

Gene symbols	Primers
IL1β	Forward 5′- GCAACTGTTCCTGAACTCAACT − 3′ Reverse 5′- ATCTTTTGGGGTCCGTCAACT − 3′
iNOS	Forward 5′- GACCATGGAGCATCCCAAGTACG − 3′ Reverse 5′- CTCGGTGCCCATGTACCAACC − 3′
Tnfα	Forward 5′- GTGGAACTGGCAGAAGAG − 3′ Reverse 5′- GCCATAGAACTGATGAGAGGG − 3′
Ym1	Forward 5′- GCTCATTCTTGTCACAGGTCTG − 3′ Reverse 5′- GGCATAGATCAGGTGAGTACAC − 3′
Arg1	Forward 5′- GGGTGGAGACCACAGTCTGG − 3′ Reverse 5′- GAAAGGACACAGGTTGCCCAT − 3′
Fizz1	Forward 5′- GGAACTTCTTGCCAATCCAGCTAAC − 3′ Reverse 5′- CCCAAGATCCACAGGCAAAGCC − 3′
GAPDH	Forward 5′- GTGGAAGGGCTCATGACCAC − 3′ Reverse 5′- GACCTTGCCCACAGCCTTGG − 3′
KIF1A	Forward 5′- GTCCTACTCTGTGGAGGTCAG − 3′ Reverse 5′- GGATGTCATTGTAGGAGGTTACAG − 3′
KIF1B	Forward 5′- CCAAAGGAACTCGATTAAAGGAAGG − 3′ Reverse 5′- GCCACCGAGATTTTCTCGAAG − 3′
KIF3A	Forward 5′- TCCCTGACAAGAAGGAGAGAGACC − 3′ Reverse 5′- ATCTTTGGCCTTGCTTTCCCCTTG − 3′
KIF17	Forward 5′- GGAGAAGATGCAGAGGAAGCTC − 3′ Reverse 5′- CCAGGAGTTGCTGGAAGAGC − 3′
KIF13A	Forward 5′- CTCTCCTAAGCCAAGAGGACTC − 3′ Reverse 5′- CACTGAAGTCAGCGAACTCCAC − 3′

### Statistical analyses

Details of the statistical analyses are described in the figure legends. Paired and Welch’s *t*-tests were used to compare two independent samples. A one-way analysis of variance (ANOVA) with Bonferroni’s post hoc test was used for multiple comparisons. The significance level was set at *P* < 0.05. The GraphPad Prism 5 software (GraphPad Software Inc., CA, USA) was used for all statistical analyses.

## Results

### Establishing the manipulation of primary cultured microglia

**Fig. 1:**
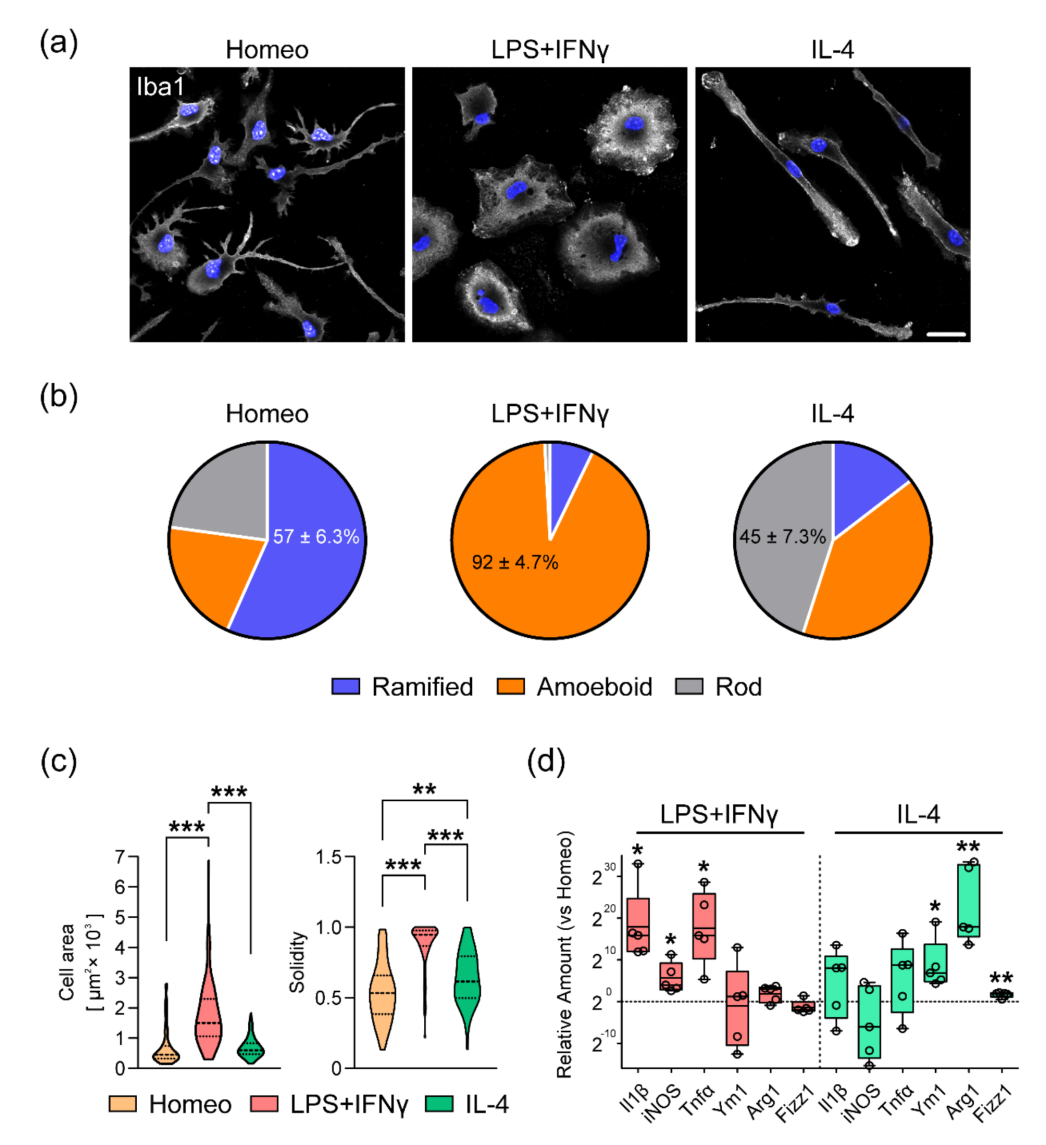
Morphological characterization and gene expression of primary microglia for each condition. (**a**) Representative images showing Iba1-immunostained microglia in the homeostatic (Homeo) condition following LPS + IFNγ or IL-4 stimulation. Cells were counterstained with DAPI. Scale bar, 20 μm. (**b**) Pie charts demonstrating the distribution of cell morphology in the Homeo, LPS + IFNγ, and IL-4 conditions. Ramified, amoeboid, and rod cells were enriched in the Homeo (57 ± 6.3%), LPS + IFNγ-stimulated (92 ± 4.7%), or IL-4-stimulated (45 ± 7.3%) conditions, respectively. *n* = 4 independent experiments. (**c**) Violin plots showing comparisons of cell area (left panel) and solidity coefficient (right panel) for each condition. *P* < 0.001, one-way ANOVA; **P < 0.01, ***P < 0.001, Bonferroni’s post hoc comparison. *n* = 68 cells for each condition for four independent experiments. (**d**) Real-time qPCR revealed increased expression of pro-inflammatory genes such as *Il1β*, inducible nitric oxide synthase (*iNOS*), and tumor necrosis factor alpha *(Tnfα*), and anti-inflammatory genes such as *Ym1*, Arginase 1 (*Arg1*), and *Fizz1* upon LPS + IFNγ or IL-4 challenges, respectively. Gene expression levels of *Gapdh* were used as internal control. In the box plots, the central line represents the median, the edges of the box represent the 25th and 75th percentiles, and the whiskers represent the minimum to the maximum. *n* = 5 independent cultures. *P < 0.05, **P < 0.01, Paired *t*-test versus Homeo

To investigate the relevance of the intracellular transport system in homeostatic and reactive microglia, we first established the primary microglia culture. It has recently been reported that the phenotype of ramified microglia is maintained by growth factors secreted by astrocytes [8, 9]. Therefore, using a medium that contained such growth factors (e.g., TGF-β2 and IL-34), we purified primary microglia and confirmed that most of the cultured cells were Iba-1, a microglial marker [9], positive (Fig. 1a). Additionally, to induce microglia polarization toward different reactive states, primary microglia were challenged with either LPS + IFNγ or IL-4, which have been shown to steer microglia toward an activated or alternatively activated state, respectively. We then classified morphology according to the number of processes and ramifications following Iba-1 staining (Fig. 1b and Additional file 3: Fig. S3): ramified (≥ 3 processes and ramifications; the cells classified into ramified are immature because of the short duration of culture), amoeboid (≤ 1 process), or rod (2 processes). The distribution of morphology classification revealed that the ramified cells were enriched in untreated microglia (57% ± 6.3%). Upon LPS + IFNγ challenge, the amoeboid cells represented a large majority of cells (92% ± 4.7%), whereas microglia predominantly acquired a rod-shape morphology (45% ± 7.3%) when cells were challenged with IL-4 (Fig. 1a and b). To further examine whether this classification corresponds to the phenotype of homeostatic and reactive microglia, as assessed by cell area, solidity, and the expression levels of their signature activation genes, we analyzed the morphology of Iba-1-positive cells and conducted RT-qPCR (Fig. 1c and d). The cell area of microglia in the LPS + IFNγ condition was the largest, and those in the homeostatic and IL-4 conditions were similar. Solidity, an index of cell roundness, in the LPS + IFNγ condition was nearly 1 (0.88 ± 0.020) and those in the homeostatic condition were significantly lower than the other two conditions (Fig. 1c). In line with these findings, the RT-qPCR revealed increased expression levels of pro-inflammatory and anti-inflammatory genes upon LPS + IFNγ or IL-4 challenge, respectively (Fig. 1d). These results are consistent with a previous report [9], which suggested that we successfully established a method to manipulate the primary microglial substate without contradiction.

### *Kif3a, Kif17*, and *Kif13a* mRNA are potentiated in microglia stimulated by IL-4, and intact KIF17 is essential for a rod-shape morphology

**Fig. 2:**
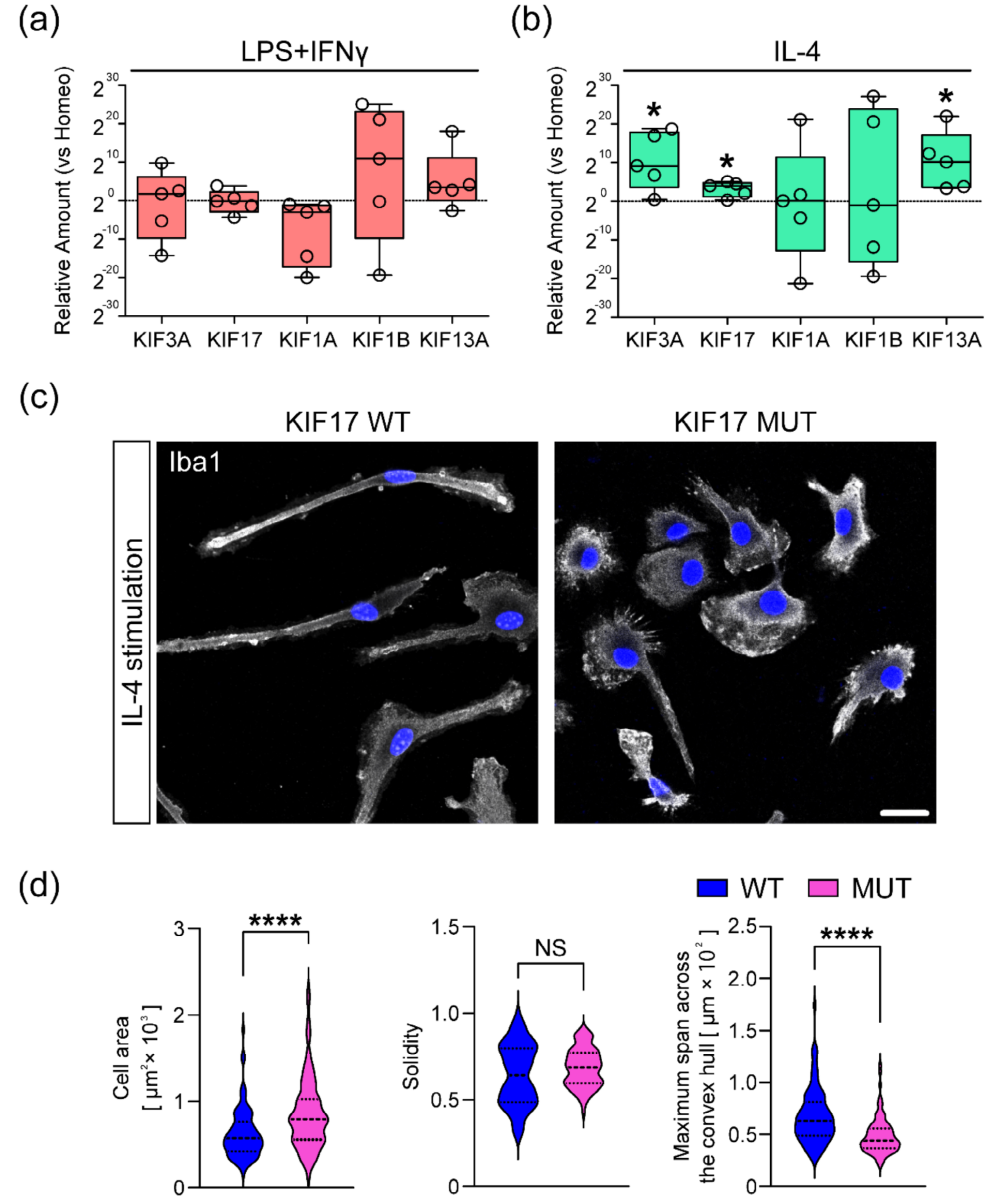
Analyses of expression levels of psychiatric disorder-related *Kifs* mRNA and morphology of alternatively activated microglia which have a truncating mutation of *Kif17*. (**a** and **b**) Quantification of real-time qPCR. *Kif3a*, *Kif17*, and *Kif13a* mRNA expression were potentiated in alternatively activated microglia. Expression levels did not significantly change in activated microglia, and neither *Kif1a* nor *Kif1b* mRNA changed in either state. *n* = 5 independent cultures. *P < 0.05, Paired *t*-test versus Homeo. (**c**) Representative images showing Iba1-immunostained microglia derived from KIF17 WT and MUT mice which were stimulated by IL-4. Cells were counterstained with DAPI. Scale bar, 20 μm. (**d**) Violin plots showing comparisons of cell area (left panel), solidity coefficient (center panel) and MSACH (right panel) for each genotype. ****P < 0.0001, NS, *P* ≥ 0.05, Welch’s *t-*test. *n* = 90 cells for two independent experiments

Next, we examined the alteration of *Kifs* gene expression between homeostatic and reactive microglia. As a candidate for a crucial *Kifs* gene that is likely related to microglia polarization, we selected psychiatric disorder-related KIFs (KIF3A, KIF17, KIF1A, KIF1B, and KIF13A) [15–21]. The relative amounts of all *Kifs* mRNA did not significantly change in microglia upon LPS + IFNγ stimulation (Fig. 2a). However, the relative amounts of *Kif3a*, *Kif17*, and *Kif13a* mRNA were significantly potentiated in microglia upon IL-4 stimulation (Fig. 2b). In contrast, the amounts of *Kif1a* and *Kif1b* mRNA did not significantly change even upon IL-4 stimulation (Fig. 2b). Additionally, to investigate the relevance of *Kif17*, one of the potentiated genes, to steering microglia toward the alternatively activated state, we established *Kif17* mutation mouse line which possessed a nonsense truncating mutation discovered from a patient with SCZ [19] and conducted Iba1-immunostaining using microglia from homozygous mutated mice (KIF17 MUT) stimulated by IL-4 (Fig. 2c). We observed the disrupted rod-shape morphology of KIF17 MUT microglia compared with KIF17 WT, assessed by cell area, solidity, and MSACH. Solidity was not significantly changed, whereas cell area and MSACH, a valuable parameter to analyze the morphology [24], of KIF17 MUT were larger and shorter than KIF17 WT, respectively (Fig. 2d). These results suggested that the mutation of *Kif17* induced the disruption of rod-shape morphology.

## Discussion

In this study, we successfully established the manipulation of primary cultured microglia polarization. Our results suggest that the potentiation of specific motor proteins was required to steer microglia toward alternatively activated state, and KIF17 transport is essential for maintain the rod-shape morphology of alternatively activated microglia as the KIF17 mutation introduced here truncates the cargo-binding region [19]. Specifically, alternatively activated microglia are primarily involved in providing neuroprotective support during inflammation or following brain injury [7]. Although IL-4 stimulation to our microglia culture consistently induced features of alternatively activated microglia, the RT-qPCR results showed a slightly discrepant signature activation gene expression profile compared with a previous report (Fig. 1d) [9]. We speculate that this was because the previous study used an astrocyte-conditioned medium to maintain the population of ramified cells, whereas we applied growth factors to microglia, as performed previously [8].

KIF3A and KIF17 that specifically transport NMDAR subunit 2 A and 2B in neurons, respectively [15, 25], are involved in the pathogenesis of psychiatric disorders [15, 19]. A mouse model of impaired NMDAR subunit 2 A transport due to a partially disrupted KIF3 complex exhibits SCZ-like features [15]. Dysfunctional microglia shown in Fig. 2 may be the cause of SCZ pathogenesis in a patient with the KIF17 mutation [19]. Furthermore, KIF13A transports the 5HT_1A_ receptor (5HT_1A_R), a serotonin receptor family protein, in hippocampal neurons, and disrupting this transport leads to anxiety-related behavioral phenotypes [21]. It has been indicated that excessive microglial activation is involved in SCZ and depression [26–30]. Although microglia express multiple receptors, including NMDAR and 5HT_1A_R, to monitor the surrounding neuronal microenvironment [31, 32], motor proteins may function via mechanisms distinct from those of neurons. Additional studies are required to gain further insight into the mechanism underlying microglial intracellular transport.

### Limitations

In this study, we used primary microglia cultures to examine the morphology and the expression levels of a specific mRNA in a pure microglia cohort. However, as resident cells of the brain, microglia continuously receive multiple signals from neurons, astrocytes, and other cells, beyond those received from the culture-conditioned medium. Therefore, we acknowledge that the interpretation of our data in vitro is limited.

## Electronic supplementary material

Below is the link to the electronic supplementary material.

Supplementary Material 1

Supplementary Material 2

Supplementary Material 3

## Data Availability

All data generated and analyzed in the current study are present in this published article. Additional data related to this article are available from the corresponding author on request.
