# Supplementary material for: Gene expression of psychiatric disorder-related kinesin superfamily proteins (Kifs) is potentiated in alternatively activated primary cultured microglia
Source: BMC Res Notes. 2025 Jan 30;18:44. doi: 10.1186/s13104-024-07078-y (PMC11783738; doi:10.1186/s13104-024-07078-y)
Supplement: Supplementary file 2 — Supplementary Material 2 [file 13104_2024_7078_MOESM2_ESM.pdf]

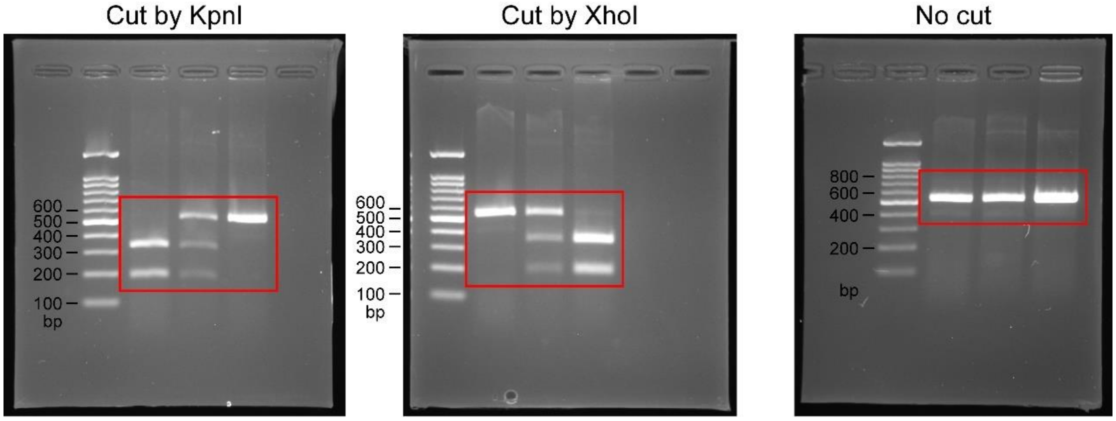

Fig. S2. Full-size uncropped images of the gel bands presented in Fig. S1c. The red boxes indicate the area presented in the figure.
