# Supplementary material for: Gene expression of psychiatric disorder-related kinesin superfamily proteins (Kifs) is potentiated in alternatively activated primary cultured microglia
Source: BMC Res Notes. 2025 Jan 30;18:44. doi: 10.1186/s13104-024-07078-y (PMC11783738; doi:10.1186/s13104-024-07078-y)
Supplement: Supplementary file 3 — Supplementary Material 3 [file 13104_2024_7078_MOESM3_ESM.pdf]

Ramified

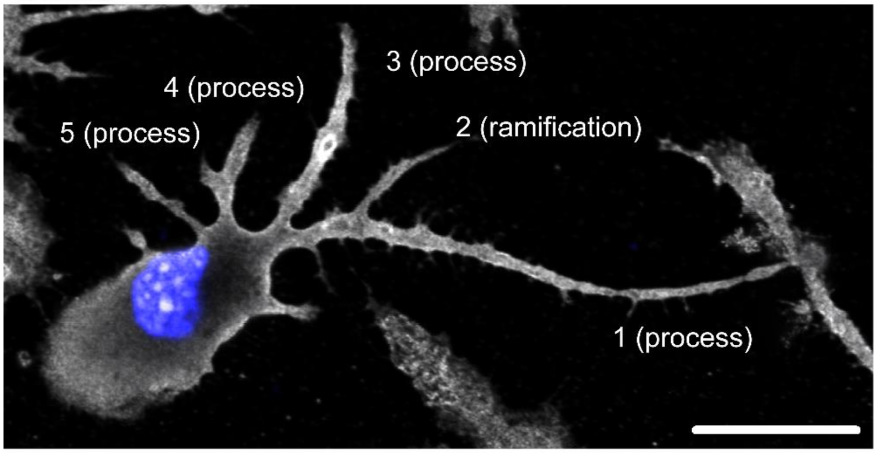

Amoeboid

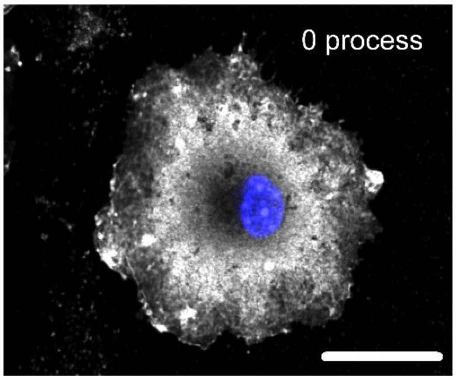

Rod

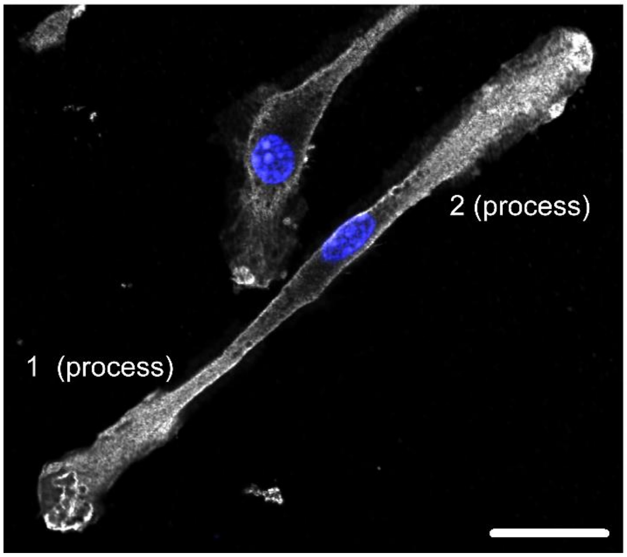

Fig. S3. How to count processes and ramifications for the classification of primary cultured microglia. The morphologies of cultured microglia were classified according to the number of processes and ramifications following Iba-1 staining. In this classification, processes and ramifications were counted equally. A linear structure longer than the maximum distance between two points across the nucleus stained with DAPI was defined here as a process or a ramification. Each microglia in high-magnified images presented here has 5 (top panel), 0 (middle panel), 2 (bottom panel) processes and ramifications. Scale bars, 20 μm.
