# Supplementary material for: Gene expression of psychiatric disorder-related kinesin superfamily proteins (Kifs) is potentiated in alternatively activated primary cultured microglia
Source: BMC Res Notes. 2025 Jan 30;18:44. doi: 10.1186/s13104-024-07078-y (PMC11783738; doi:10.1186/s13104-024-07078-y)
Supplement: Supplementary file 1 — Supplementary Material 1 [file 13104_2024_7078_MOESM1_ESM.pdf]

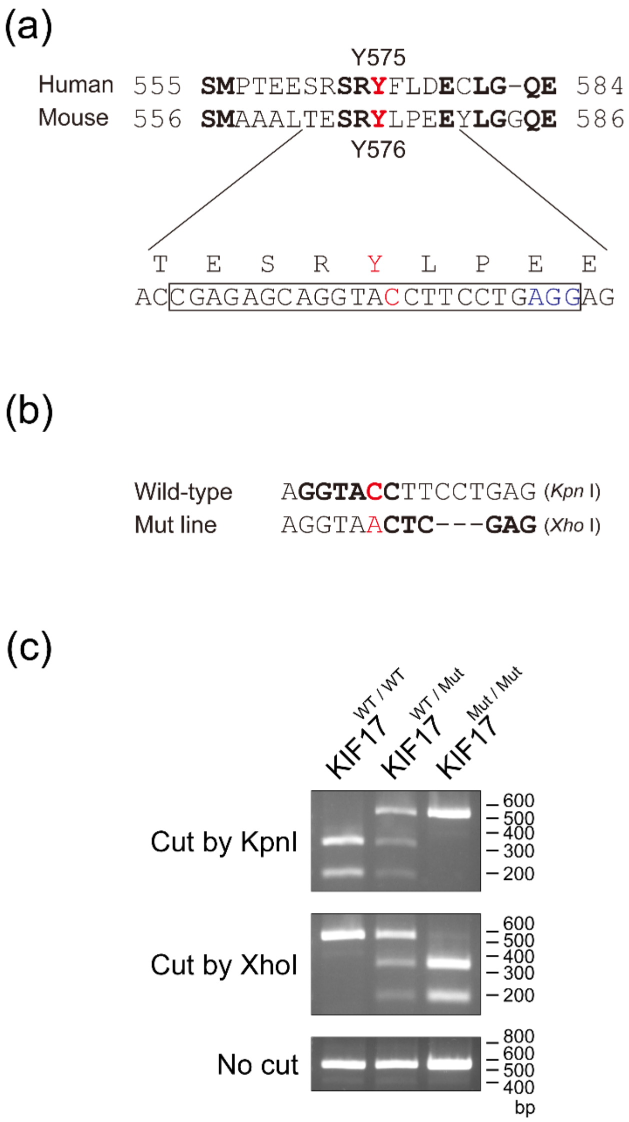

Fig. S1. Generation of *Kif17* mutation mouse line. (**a**) Comparison of human and mouse KIF17 sequences. The amino acid corresponding to tyrosine mutated in a SCZ patient is Y576 in mouse KIF17 sequence, which turns into stop codon. (**b**) The strategy of facilitating the detection of genotypes. We designed to contain XhoI restriction site downstream of the mutation. (**c**) Genotyping results of DNA from the mouse tails of the indicated genotypes. Each DNA was amplified by the PCR and subsequently digested by KpnI and XhoI.
